# High-Brightness Blue Polariton Organic Light-Emitting Diodes

**DOI:** 10.1021/acsphotonics.3c01610

**Published:** 2024-04-15

**Authors:** Julia Witt, Andreas Mischok, Francisco Tenopala Carmona, Sabina Hillebrandt, Julian F. Butscher, Malte C. Gather

**Affiliations:** † Humboldt Centre for Nano- and Biophotonics, Department of Chemistry, University of Cologne, Greinstr. 4-6, 50939 Cologne, Germany; ‡ Organic Semiconductor Centre, SUPA, School of Physics and Astronomy, University of St Andrews, North Haugh, St Andrews KY16 9SS, United Kingdom

**Keywords:** Polariton OLED, OLED, strong light−matter coupling, microcavity, BSBCz

## Abstract

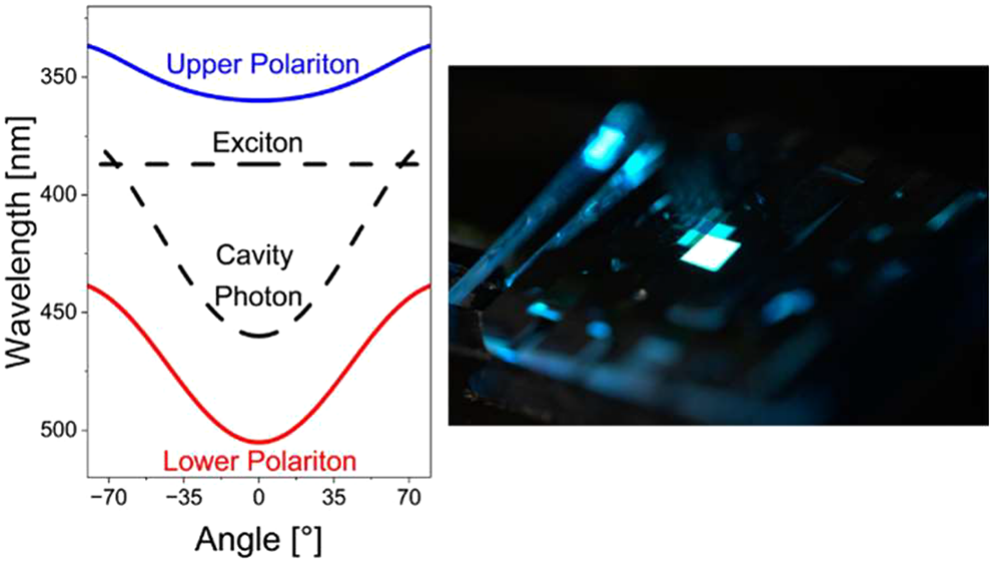

Polariton organic light-emitting diodes (POLEDs) use strong light-matter coupling as an additional degree of freedom to tailor device characteristics, thus making them ideal candidates for many applications, such as room temperature laser diodes and high-color purity displays. However, achieving efficient formation of and emission from exciton-polaritons in an electrically driven device remains challenging due to the need for strong absorption, which often induces significant nonradiative recombination. Here, we investigate a novel POLED architecture to achieve polariton formation and high-brightness light emission. We utilize the blue-fluorescent emitter material 4,4′-Bis(4-(9H-carbazol-9-yl)styryl)biphenyl (BSBCz), which exhibits strong absorption and a highly horizontal transition-dipole orientation as well as a high photoluminescence quantum efficiency, even at high doping concentrations. We achieve a peak luminance of over 20,000 cd/m^2^ and external quantum efficiencies of more than 2%. To the best of our knowledge, these values represent the highest reported so far for electrically driven polariton emission from an organic semiconductor emitting in the blue region of the spectrum. Our work therefore paves the way for a new generation of efficient and powerful optoelectronic devices based on POLEDs.

## Introduction

Exciton-polaritons are bosonic quasiparticles that form by coherent interactions of electromagnetic waves with exciton oscillations. Polariton formation is often achieved by using a strong excitonic resonance in a high-quality microcavity (MC), which minimizes the losses of the system.^1^ Under these conditions of strong light–matter coupling, hybridization of photons and excitons can lead to the formation of two new eigenstates called the upper and lower polariton.^2^ Strong coupling thus allows manipulation of the light and matter components of a system in new ways and therefore enables the design of devices with a number of interesting properties and with possible applications, e.g., in transistors,^3^ communication systems,^4^ and polariton lasers.^5−11^ In addition, very recently, we have demonstrated that strong coupling and, in particular, the unique angular dispersion relation of polaritons can be used to generate angle independent emission with high color purity, which might have profound implications for the design of future displays.^12^ In order to be able to directly manipulate these systems using strong light–matter coupling, efficient polariton OLEDs must be developed.

In organic semiconductors, polaritons are highly stable, even at room temperature, which is due to the relatively large exciton binding energies in these materials. As a result, polaritons in organic materials have been extensively studied and are of particular relevance for practical applications of strong light–matter coupling at room temperature,^13,14^ for example in OLEDs^12^ and organic photovoltaics (OPVs).^15^ So far, the majority of studies on polaritons in organic semiconductors rely on optical excitation to generate polaritons. However, direct electrical excitation of polaritons is of great interest for work toward an electrically pumped organic polariton laser diode. While efficient electrical generation of polaritons at green and red wavelengths has been achieved (up to 10% external quantum efficiency (EQE)),^12,16^ the development of efficient and bright devices emitting blue light is particularly difficult (with current devices reaching no more than 0.1% EQE)^17^ due to the high singlet state energies involved, which can lead to molecular degradation and Joule heating. Irrespective of the color of emission, upon electrical excitation, the majority of the generated excitons have triplet character, which is a direct result of spin statistics. For fluorescent emitter materials, this reduces the efficiency of emission. Devices based on triplet-harvesting materials can overcome this efficiency loss but often suffer from substantial exciton quenching and as a result a significant roll-off in EQE with increasing current.^18,19^ In combination, these issues often lead to limited efficiency, brightness, and stability of polaritonic organic light-emitting diodes (POLEDs).^20,21^ The principal challenge for the development of POLEDs is to develop materials that provide sufficiently strong absorption to reach the strong coupling regime while also showing a high photoluminescence quantum yield (PLQY). We recently have shown that this issue can be mitigated by the addition of assistant strong-coupling layers within the transport layers of a POLED. If positioned correctly, this strong-coupling layer does not interfere with the emission process and thus facilitates efficient polariton formation without reducing device efficiency.^12^ Others have reported architectures relying on coupled or intracavity radiative pumping.^21,22^

In this paper, we report on the use of the blue fluorescent emitter molecule bis[(*N*-carbazole)styryl]biphenyl (BSBCz) in a simple device architecture that achieves both efficient operation and strong light–matter coupling. Our device does not require the use of an assistant strong coupling layer or intracavity pumping, which will likely be beneficial for efforts to realize an electrically pumped polariton laser. BSBCz is an ideal candidate for this task, as it maintains high PLQY, even in highly doped films, and features a highly horizontal transition-dipole orientation (TDO), which facilitates efficient coupling of its emission to the vertical cavity mode.^23^ Our optimized device architecture was developed by starting from a weakly coupled device with no polariton formation. We report two device configurations exhibiting strong light–matter interaction, namely, a bottom- and a top-emitting architecture. The top-emitting architecture is of significant interest for future (flexible) displays and high-current applications, both of which benefit from the ability to use opaque substrates.^24^ Our devices achieve a record external quantum efficiency (2.2%) and brightness (>20,000 cd/m^2^) among the polariton OLEDs without an assistant strong coupling layer reported to date.

## Methods

### OLED Fabrication

All of the OLEDs were produced via thermal evaporation. Metals and organic materials were heated in a vacuum chamber (*EvoVac, Angstrom Engineering*) at a base pressure of 10^–7^ mbar. The layers were patterned through shadow masks, creating active areas of 4 mm^2^ defined by the anode–cathode overlap. The evaporation rates and layer thicknesses were monitored by quartz crystal microbalances.

All organic materials were obtained from *Lumtec* in sublimed grade and used without further purification. After evaporation the devices were encapsulated in a nitrogen-filled glovebox. Getter-embedded (*Dynic*) glass lids were attached by an UV-curable epoxy glue (*Norland Optical Adhensive 61*).

### OLED Characterization

Current-density and luminance characterization were performed with a spectrometer (*OceanHDX*, *Ocean Optics*), a source-measurement unit (*2450 SourceMeter, Keithley*), and an amplified Si photodiode (*PDA100A, Thorlabs*) connected to a multimeter (*2100, Keithley*). Angle resolved spectra were recorded with a custom setup as described in *Archer* et *al.*,^25^ using constant current operation typically at 3.5 mA. For better contact, silver paste (*PLANO GmbH*) was applied to the contact pads on the devices prior to the measurements.

### Emission Dipole Orientation Measurements

ARPL measurements were carried out on a setup described elsewhere.^25^ The data was fitted to optical models based on the transfer-matrix method using anisotropic optical constants obtained from variable-angle spectroscopic ellipsometry. The emitter dipole orientation and layer thickness were used as free fitting parameters, and the orientation values were obtained from the best fits to the ARPL data.^26^

### Reflection Measurements

Reflection measurements were performed using variable-angle spectroscopic ellipsometry (*VASE M2000, J. A. Woollam*) by measuring changes in intensity and polarization of light reflected at different angles of incidence (45°–78°). The data was analyzed via *CompleteEase* software (*J. A. Woollam*) and *Origin 2022b*. To compare the high-angle results with the light dispersion at lower angles, simulations were performed using transfer-matrix calculations (0°–78°). Simulations of two coupled oscillators were adjusted to the measured data to analyze the coupling strengths.

## Results

**Figure 1:**
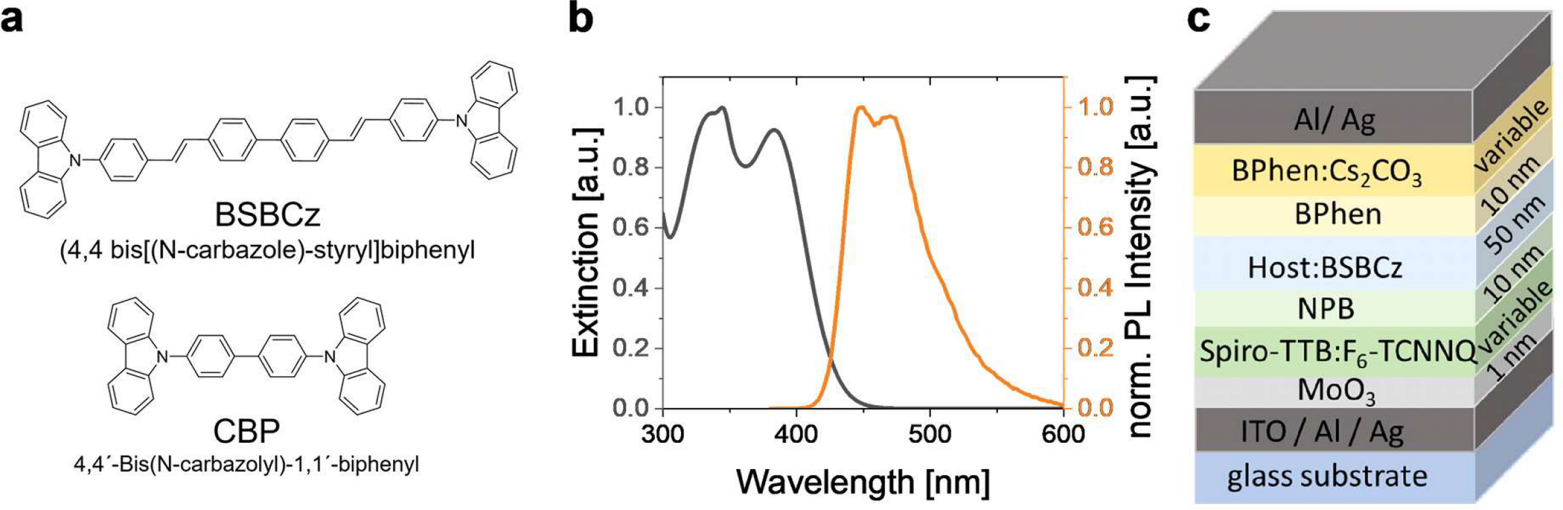
**(a**) Chemical structures of the BSBCz emitter and the CBP host used in this study. (**b**) Extinction (gray) and photoluminescence (orange) spectra of a 50 wt % CBP:BSBCz thin film. (**c**) General device architecture of the blue-emitting POLEDs developed here. The host material was varied between TCTA, mCBP, and CBP, in each case using 50 wt % doping of BSBCz. The material and thickness of the electrodes as well as the thickness of the BPhen:Cs_2_CO_3_ (20 wt %) electron transport layer (ETL) and the Spiro-TTB:F_6_-TCNNQ (4 wt %) hole transport layer (HTL) were also varied. The specific layer architectures for each device are listed in the Supporting Information (Tables S2 and S3).

Figure 1a shows the molecular structure of BSBCz. BSBCz has been shown to exhibit nearly balanced bipolar charge carrier mobility (μ_e-_ ≈ 3 × 10^–4^ cm^2^/(V s), μ_h+_ ≈ 7 × 10^–4^ cm^2^/(V s)), high photoluminescence quantum efficiency (≈77%), as well as a short fluorescence lifetime (≈1 ns), even in a neat film or when mixed into a host material at high concentration.^23,27,28^ Due to its strong excitonic absorption, which peaks at around 370 nm, BSBCz is a good candidate for strong light–matter coupling (Figure 1b). Its photoluminescence spectrum shows maxima at 448 nm, 470 nm, and 512 nm, which correspond to the vibronic 0–0, 0–1, and 0–2 transitions, respectively.^29,30^ Furthermore, pure BSBCz is strongly horizontally oriented when deposited as a thin film,^31,32^ which will be beneficial to the outcoupling efficiency of OLEDs based on it and can facilitate efficient strong coupling to vertical cavity modes.

Mixing an emitter material into a host can increase the chemical, physical, and electrical stability of devices. However, as the addition of host materials can also affect the molecular orientation of the emitter,^32^ we first performed measurements of the TDO for a series of hosts and doping concentrations (Supporting Information Figure S1 and Table S1), finding that for 4,4′-bis(*N*-carbazolyl)-1,1′-biphenyl (CBP) as host material in particular, a very high horizontal TDO of 95% was achieved.

Next, we compared devices with emissive layers (EMLs) based on BSBCz doped into either CBP, tris(4-carbazoyl-9-ylphenyl)amine (TCTA), or 3,3′-di(9H-carbazol-9-yl)-1,1′-biphenyl (mCBP) to investigate which host provides the best performance and stability in an OLED. For this test and all other devices in this study, we used an emissive layer thickness of 50 nm at a doping concentration of 50 wt % BSBCz to ensure our devices operate in the strong light–matter coupling regime. We further employed a p-i-n device architecture, embedding the emissive layer between charge blocking layers and electrically doped charge transport layers (Figure 1c).^33^ In detail, the device consisted of molybdenum trioxide (MoO_3_) as hole injection layer, 2,2′,7,7′*-*tetrakis(*N,N*′-di-*p*-methylphenylamino)-9,9′-spirobifluoren (Spiro-TTB) doped with 2,2′-(perfluoro-naphthalene-2,6-diylidene)dimalononitrile (F_6_-TCNNQ) (4 wt %) as hole transport layer (HTL), *N*,*N′*-di(naphthalene-1-yl)-*N,N′*-diphenylbenzidine (NPB) as electron blocking layer (EBL), the BSBCz based EML 4,7-diphenyl-1,10-phenanthroline (BPhen) as a hole blocking layer (HBL), and BPhen doped with cesium carbonate (Cs_2_CO_3_) as electron transport layer (ETL); see Supporting Information Table S2 for specific layer thicknesses. The devices feature a combination of semitransparent and opaque metal electrodes, which consist of 25 and 100 nm thick silver layers, respectively. Silver shows high reflectivity in the blue and is well suited as an electrode material, enabling ohmic charge injection into the doped charge transport layers. To guarantee formation of a smooth film, 1 nm of Al was deposited underneath the silver electrodes.^34^ The combination of semitransparent and opaque electrodes creates a Fabry–Perot microcavity, enhances the exciton–photon interaction, and facilitates strong coupling in the EML.^35^ The thickness of the organic layers was optimized by transfer matrix calculations to ensure the field maximum of the cavity mode is located in the emission layer of the OLED and thus enable maximum light–matter interaction as well as efficient outcoupling of light.^36^

The device characteristics for the different host materials were analyzed and compared (Supporting Information Figure S2). While the mCBP containing device reached a particularly high EQE of 2.5%, devices with a CBP host yielded the best overall performance, with the lowest turn-on voltage, highest luminance, and best stability at high voltages; CBP was hence used for all further devices in this study. (Turn-on voltage was defined as the voltage at which the rapid increase in current density starts in the *j*–*V* curve.)

To make the device compatible with opaque substrates, as is required for example to maximize thermal conductivity in efforts toward electrically pumped lasers, the architecture of the device was not only adjusted for a bottom-emitting design (BE-POLED) but also for a top-emitting cavity design (TE-POLED).^24^ BE-POLED and TE-POLED were fabricated by making either the anode or the cathode semitransparent. A third (reference) device was fabricated using indium tin oxide (ITO) as transparent electrode to compare its characteristics to the cavity devices. Only the TE-POLED comprised the MoO_3_ hole injection layer, because the other devices did not show problems with stability or charge injection.^37^

**Figure 2:**
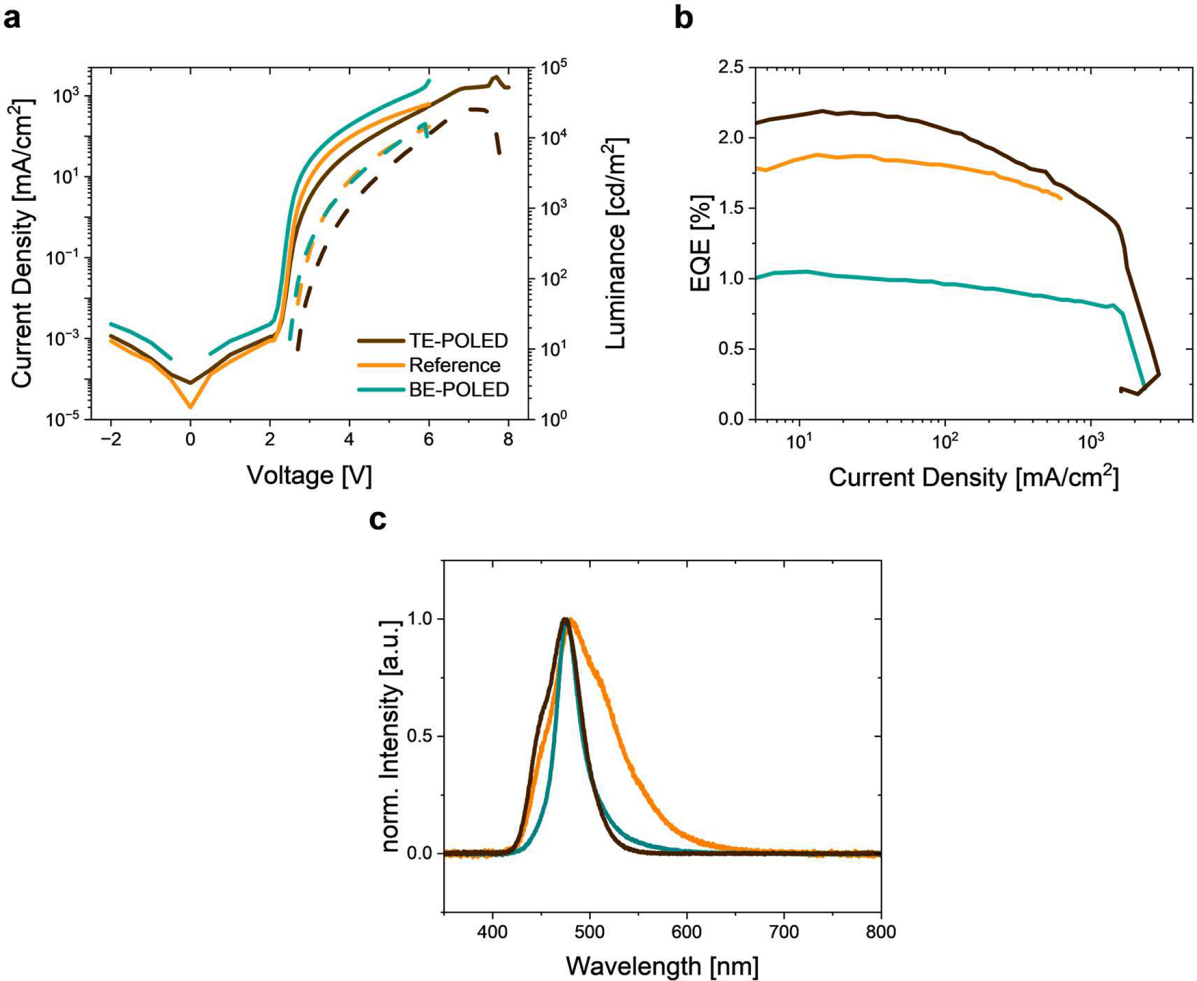
Characteristics of the reference OLED, BE-POLED, and TE-POLED. (**a**) Current density (solid lines) and luminance (dashed lines) over applied voltage. (**b**) EQE over current density. (**c**) Electroluminescence spectra.

The *jVL* characteristics of the BE-POLED, TE-POLED, and reference OLED are shown in Figure 2. Additional data and statistics are given in Supporting Information Figure S3 and S4. To ensure the comparability of the three device types, the angle-resolved light emission was taken into account for evaluation (Supporting Information, Figure S5). The reference OLED shows a turn-on voltage of 2.1 V and reaches a luminance of 7219 cd/m^2^ at 5 V (Figure 2a). It reaches an EQE of 1.9% with relatively minor roll-off across the measured luminance range. The *jVL* characteristics of the cavity BE-POLED indicate a similar behavior, with a luminance of 6871 cd/m^2^ at 5 V but a lower EQE of 1.0%, which results from the spectral shift of the peak of its emission spectrum to 473 nm. Nevertheless, both devices show a luminance of above 10,000 cd/m^2^ at higher voltages, which is high when compared to most previous demonstrations of POLEDs,^38,16,39^ in particular for a blue-emitting device.^17^ At a current density of 1651 mA/cm^2^, we observe complete degradation of the BE-POLED, which manifests itself as a strong increase of current density and drop in the luminance (Figure 2b). This breakdown current density is higher than the maximum value of 620 mA/cm^2^ that the reference-state OLED reaches. The difference in the maximum current density tolerated by the two devices probably results from the different conductivities of the anodes used, with the ITO anode in the reference OLED showing lower conductivity than the 1 nm Al and 25 nm Ag anodes in the BE-POLED.

The change from the BE-POLED to the TE-POLED configuration was achieved by adjusting the thickness of the two silver mirrors and the transport layers. To optimize light outcoupling, a 40 nm thick layer of NPB was also added on top of the cathode contact (details on the device stack can be found in Supporting Information Table S3). The TE-POLED shows lower luminance (3940 cd/m^2^) and current density (167 mA/cm^2^) at 5 V than the reference OLED and BE-POLED devices. However, it also reaches a higher luminance of over 20,000 cd/m^2^ at higher voltages. The device fails at an applied voltage of 7.1 V, at which point the current density reaches 1589 mA/cm^2^, which is similar to the maximum current density reached by the BE-POLED. However, the maximum EQE of the TE-POLED approximately doubles relative to that of the BE-POLED, reaching 2.2%. We attribute this difference to a more optimal tuning of the cavity spectrum to the photoluminescence spectrum of the 50 wt % CBP:BSBCz thin film for the TE-POLED (Figure 2c). Another reason could be the presence of the additional layer of MoO_3_, which might facilitate better hole injection into the device.

**Figure 3:**
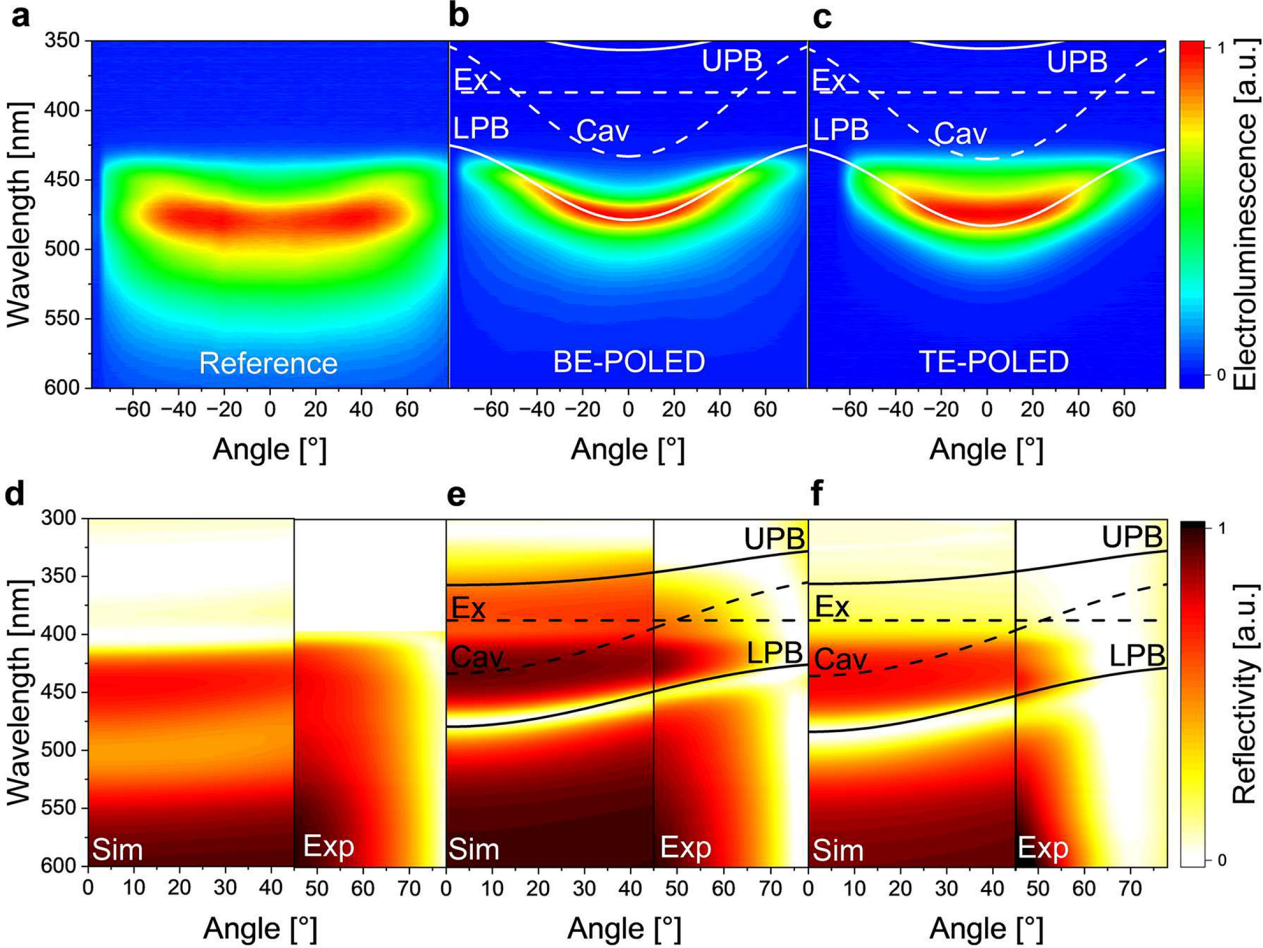
(**a**–**c**) Angle-resolved electroluminescence measurements of (**a**) the reference OLED, (**b**) the BE-POLED, and (**c**) the TE-POLED. The overlaid dashed lines show the exciton energy (Ex) and the cavity mode (Cav), and the solid lines depict the lower (LPB) and upper polariton branch (UPB) as predicted by the oscillator model with an exciton energy corresponding to 387 nm and cavity modes at 433 nm (BE-POLED) and 435 nm (TE-POLED). (**d**–**f**) Angle-resolved reflectivity measurements of (**d**) the reference OLED, (**e**) the BE-POLED, and (**f**) the TE-POLED. The left side of panels **d–f** shows spectra from transfer matrix simulations (Sim), and the right side shows the experimental data (Exp). Extended simulated data (up to 78°) can be found in Supporting Information Figure S9.

To confirm the presence of strong coupling and polariton generation in our OLEDs, we performed angle-resolved electroluminescence (AREL) and reflectivity measurements. Figure 3 shows the results and compares them to the expected positions of the cavity mode, the lower polariton branch (LPB), and the upper polariton branch (UPB), as well as to the exciton binding energy. The expected position and dispersion of the LPB and UPB were obtained using a model of two coupled oscillators^12^ with the coupling strength and the detuning of the cavity mode as free fitting parameters. The shape of the LPB was adjusted to the emitted and reflected light dispersion taken from the spectra, as the UPB is not visible in the reflection measurement (Figure 3e,f). This is due to both additional absorption of the Spiro-TTB hole transport layer that is present in the device stack and the reduced reflectivity of silver at wavelengths below 350 nm. The breakdown of the upper polariton branch might be due to high-energy absorption bands in the cavity.^40^ In addition, it has been found that the upper polariton can likely extend beyond the reflectivity range of the mirror employed (e.g., a DBR stopband;^41^ or the Ag plasma frequency), leading to either the vanishing of the UP or the creation of new modes. Additional simulations of BSBCz in an empty cavity (Supporting Information Figure S6) further indicate that the absorption in the system should be treated as inhomogeneously broadened.^42^ Furthermore, to provide additional evidence for the ability of the CBP:BSBCz mixture to support strong coupling, aluminum-clad cavities were fabricated. These show a coupling to both BSBCz and CBP excitons and the presence of an UP and middle polariton branch (Supporting Information Figures S7 and S8). The shape of the angle-resolved emission of the BE-POLED and TE-POLED follows the shape of the LPB, showing cavity-like behavior at smaller angles and exciton-like flattening at higher angles. Consistent with earlier reports on POLEDs, there was no emission from the UPB. The reference device instead shows spectrally broad emission, as expected for the weak cavity formed by the ITO anode and the Ag cathode. The coupled oscillator model predicts a Rabi splitting of *G* = 820 meV for the BE-POLED and 850 meV for the TE-POLED. This very large splitting is caused by the strong absorption of the emissive layer with high doping concentration of BSBCz (50 wt %) and the use of a metallic microcavity with relatively small mode volume.^30^ The BE-POLED shows a steeper LPB following more closely the shape of the cavity-like light dispersion. The corresponding reflection spectra confirm the position of the LPB observed in the AREL spectrum, where the TE-POLED shows a broader LP resonance. The UPB is obscured by strong absorption from the charge transport layers and the quickly diminishing reflectivity of Ag in the UV. Nevertheless, the reflection measurements show the typical light dispersion of the LPB.

## Conclusion

Due to their relatively angle-independent and narrow band emission, POLEDs show great potential for future displays. Since the LPB can be shifted depending on the coupling strength and position of the optical modes, high color-purity, tunable, and angle-independent emission can be achieved. Generating efficient electroluminescence in these devices is also a crucial step toward any future electrically pumped organic polariton laser. However, with a few recent exceptions, POLEDs have significantly lagged behind in performance compared to standard OLEDs that operate in the weak coupling regime.

In this work, by exploiting the highly horizontal orientation of the BSBCz emitter and the fact that it maintains an exceptionally high PLQY even when doped at 50 wt % into a CBP host, we presented record brightness and efficiency blue POLEDs achieving a luminance of over 20,000 cd/m^2^. We verified that BSBCz maintains its horizontal TDO when mixed into different hosts and identified CBP as particular well suited for the fabrication of POLEDs. We compared a bottom-emitting strongly coupled cavity configuration with an ITO reference device with a weak microcavity. We also showed top-emitting devices, which in the future will allow the use of different substrates, e.g., thermally conductive substrates that can enable device operation at higher current levels than presently possible.

## Supporting Information Available

Photoluminescence and ellipsometry measurement of 50 wt % CBP:BSBCz thin layer. Orientation of BSBCz as neat film and in CBP and TCTA matrices. Specific layer thicknesses for POLEDs containing different matrix materials. JVL measurements of POLEDs with different matrix materials. Specific layer thicknesses of the reference OLED, BE-POLED, and TE-POLED. Statistics of the reference, BE-POLED, and TE-POLED. Simulations of the reflection spectra for the reference OLED, BE-POLED, and TE-POLED for 0°–78°. Reflectivity simulations of BSBCz cavities of 1st and 2nd order and an “empty” cavity 2nd order and experimental results of a 1st order BSBCz cavity. Normalized angle-resolved luminance of the reference OLED, BE-POLED, and TE-POLED. Angle-resolved transmission of 1st and 2nd order aluminum-clad cavities. (PDF)
