# Supplementary material for: High-Brightness Blue Polariton Organic Light-Emitting Diodes
Source: ACS Photonics. 2024 Apr 15;11(5):1844–50. doi: 10.1021/acsphotonics.3c01610 (PMC11100280; doi:10.1021/acsphotonics.3c01610)
Supplement: Supplementary file 1 — ph3c01610_si_001.pdf [file ph3c01610_si_001.pdf]

Supporting Information

High-brightness blue polariton organic light-emitting diodes

Julia Witt[1], Andreas Mischok[1], Francisco Tenopala Carmona[1], Sabina Hillebrandt[1], Julian F. Butscher[1,2], Malte C. Gather[1,2*].

[1]Humboldt Centre for Nano- and Biophotonics, Department of Chemistry, University of Cologne, Greinstr. 4-6, 50939 Cologne, Germany

[2]Organic Semiconductor Centre, SUPA, School of Physics and Astronomy, University of St Andrews, North Haugh, St Andrews KY16 9SS, United Kingdom

*malte.gather@uni-koeln.de

Contents

**Figure S1**	Photoluminescence and ellipsometry measurement of 50wt% CBP:BSBCz thin layer
**Table S1**	Orientation of BSBCz as neat film and in CBP and TCTA matrices
**Table S2**	Specific layer thicknesses for POLEDs containing different matrix materials
**Figure S2**	JVL measurements of POLEDs with different matrix materials
**Table S3**	Specific layer thicknesses of the reference OLED, BE-POLED and TE-POLED
**FigureS3**	Three measurements of each device stack
**FigureS4**	Statistics of the reference OLED, BE-POLED and TE-POLED
**FigureS5**	Normalized angle-resolved luminance of the reference OLED, BE-POLED and TE-POLED
**FigureS6**	Reflectivity simulations of BSBCz cavities of 1st and 2nd order, an “empty” cavity of 2nd order and experimental results of a 1st order BSBCz cavity
**FigureS7**	Polaritonic light dispersion in a 1st order Al-clad microcavity
**FigureS8**	Polaritonic light dispersion in a 2nd order Al-clad microcavity
**FigureS9**	Simulations of the reflection spectra for the reference OLED, BE-POLED and TE-POLED for 0°-78°

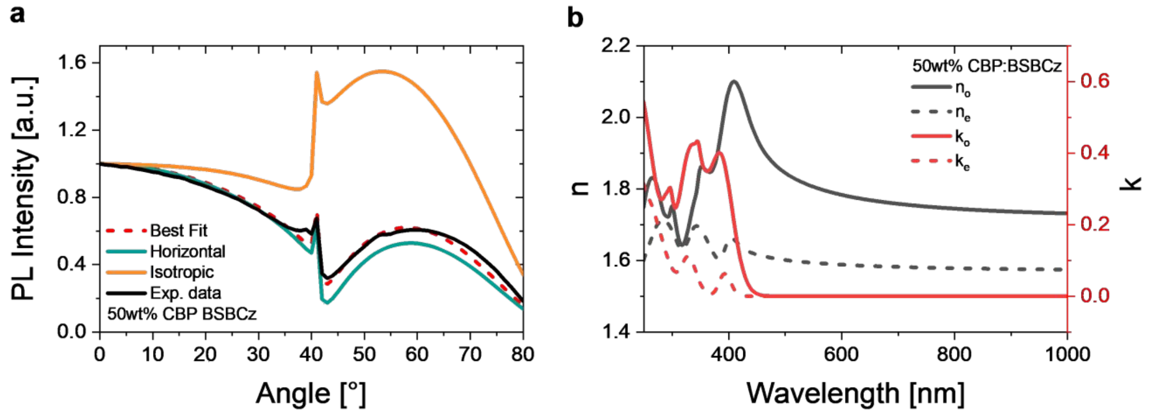

Figure S1: **a** Angle-resolved photoluminescence measurements of a 50 nm thick layer of 50wt% CBP:BSBCz. **b** Optical constants of 50wt% CBP:BSBCz measured by variable-angle spectroscopic ellipsometry.

Table S1: Measured transition-dipole orientations of BSBCz as a neat film and at different doping concentrations in CBP and TCTA. The anisotropy factor *a* describes the fraction of vertically aligned molecules, thus *a* = 0 would correspond to perfectly horizontal aligned.[26] Horizontal orientation gives the fraction of horizontally aligned transition dipoles in percent.

Host	Concentration [wt%]	a	Horizontal orientation [%]
**none**	100	0.06	94
**CBP**	50	0.05	95
**CBP**	20	0.04	96
**CBP**	8	0.05	95
**TCTA**	8	0.05	95
**TCTA**	20	0.05	95
**TCTA**	50	0.05	95
**TCTA**	80	0.05	95

Table S2: Devices structure and layer thicknesses of top-emitting POLEDs with different host materials.

Matrix Material	mCBP Layer thickness [nm]	TCTA Layer thickness [nm]	CBP Layer thickness [nm]
**ITO**	-	-	90
**Al**	1	1	1
**Ag**	25	25	25
**MoO_3**	1	1	-
**SpiroTTB:F_6TCNNQ**	110	50	115
**NPB**	10	10	10
**Matrix:BSBCz**	50	50	50
**BPhen**	10	10	10
**BPhen:Cs_2CO_3**	50	110	50
**Ag**	100	100	100

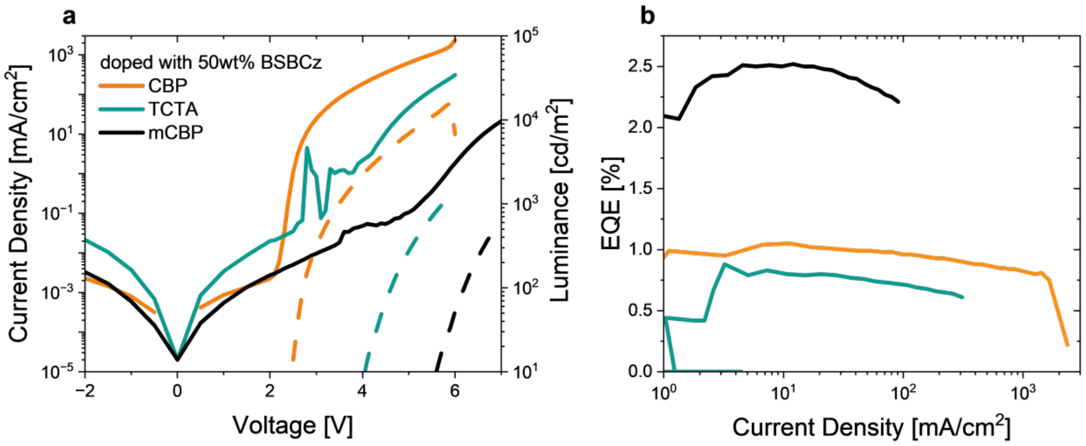

Figure S2: **a** *JVL* measurements of POLEDs with different matrix materials (mCBP, TCTA, CBP) doped with 50w% BSBCz. **b** Corresponding EQEs versus currents density.

Table S3: Devices structure and layer thicknesses of reference OLED, BE-POLED and TE-POLED.

Material	Reference Layer thickness [nm]	BE-POLED Layer thickness [nm]	TE-POLED Layer thickness [nm]
**ITO**	90	90	-
**Al**	-	1	1
**Ag**	-	25	100
**MoO_3**	-	-	1
**SpiroTTB:F_6TCNNQ**	115	115	140
**NPB**	10	10	10
**CBP:BSBCz**	50	50	50
**BPhen**	10	10	10
**BPhen:Cs_2CO_3**	50	50	20
**Al**	-	-	1
**Ag**	100	100	25
**NPB**	-	-	40

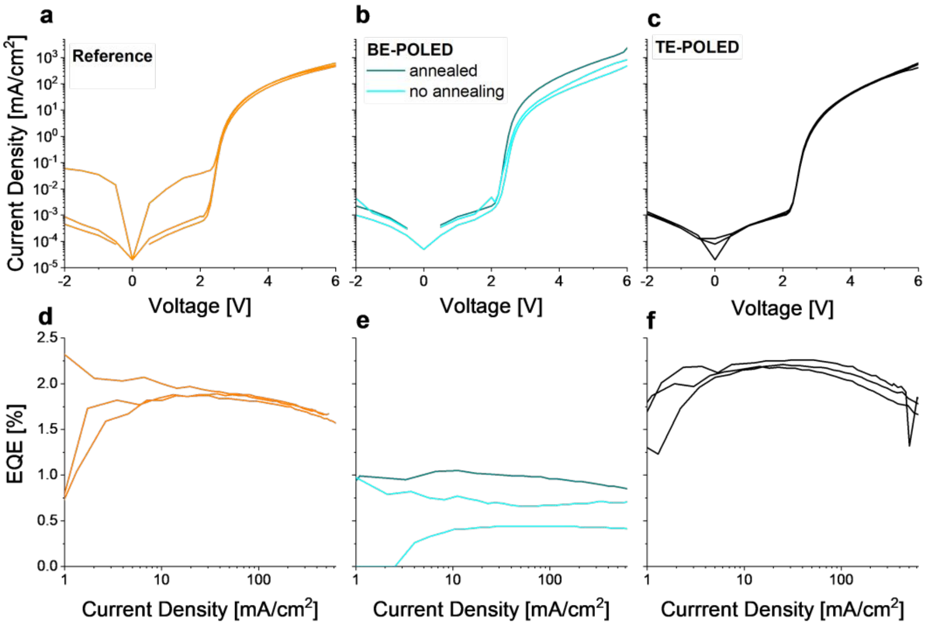

Figure S3: *J*-*V* characteristics for three OLEDs each of **a** the reference OLED, **b** the BE-POLED, and **c** the TE-POLED and the corresponding EQE measurements **d**, **e** and **f**.

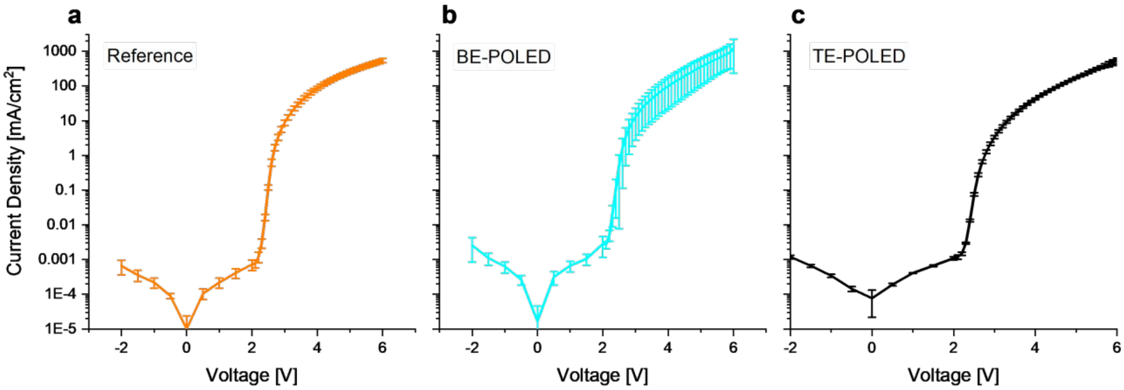

Figure S4: Mean of the *J*-*V* characteristics of the devices presented in Figure S3 with the corresponding standard deviations shown as error bars. Graph **a** depicts the reference OLED, **b** the BEPOLED, and **c** the TE-POLED.

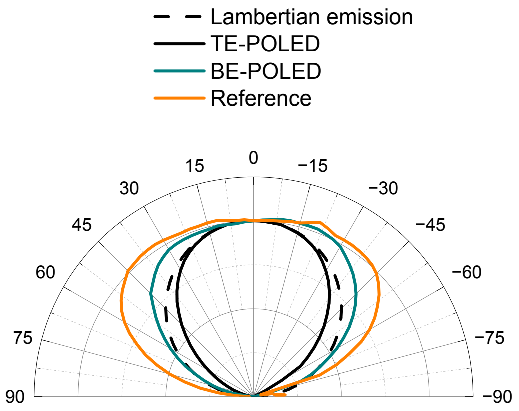

Figure S5: Normalized angle-resolved luminance of the Reference OLED, the BE-POLED, and the TE-POLED.

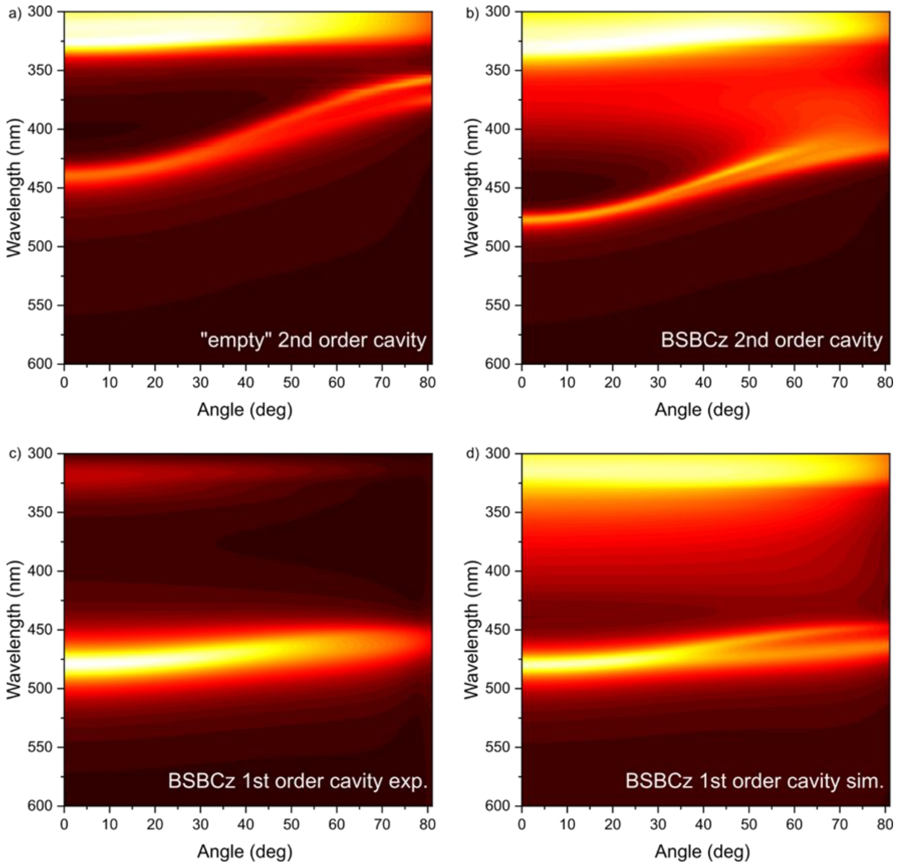

Figure S6: a,b Reflectivity simulations of a 2nd order cavity containing transparent spacers and 50 nm of a hypothetic BSBCz material without excitonic absorption ("empty" cavity, a) as cavity reference, and 50 nm of the actual BSBCz with excitonic absorption (b). c,d Experimental (c) and simulated (d) reflectivity spectra of a 1st order BSBCz cavity. The spectral redshift and flattening of the dispersion of the lower polariton mode indicate that the system is in the strong coupling regime for b-d.

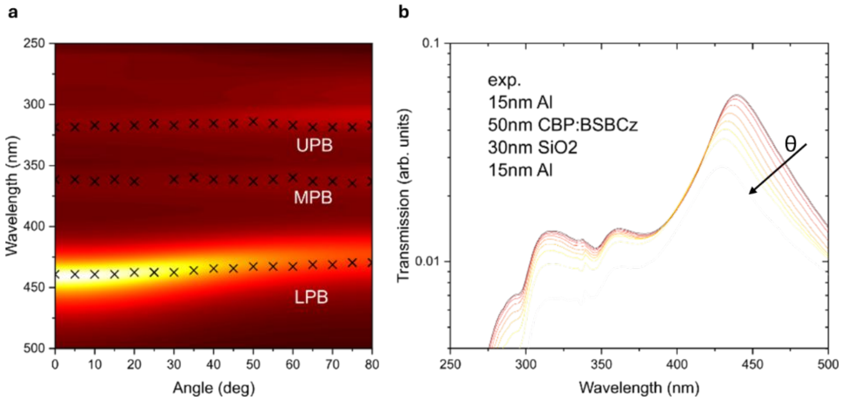

Figure S7: CBP:BSBCz cavities with Al mirrors and transparent spacers. **a** Simulated transmission for a 15nm Al|50nm CBP:BSBCz|30nm SiO2|15nm Al cavity (false color plot). The experimental transmission peaks are overlayed as crosses. **b** Measured transmission spectra at different angles for of the same cavity as discussed in **a.**

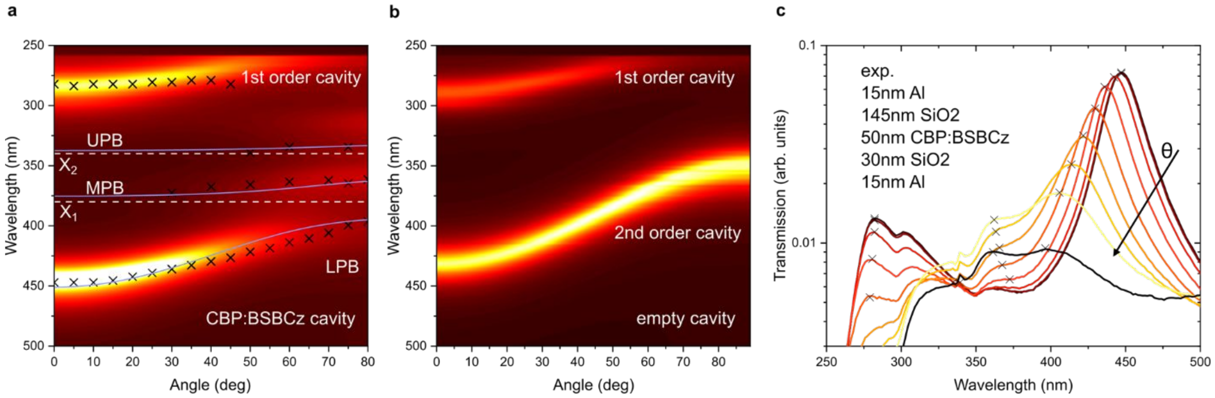

Figure S8: CBP:BSBCz cavities with Al mirrors and transparent spacers. **a** Simulated transmission for a 15nm Al|145nm SiO2|50nm CBP:BSBCz|30nm SiO2|15nm Al cavity (false color plot), comparable to the TE-POLED presented in the main manuscript. The experimental transmission peaks are overlayed as crosses. Solid lines represent the fit to a 3-coupled oscillator model, showing the appearance of lower, middle, and upper polariton branch. Dashed lines show the position of the main exciton peaks from BSBCz (X1) and CBP (X2). The extracted Rabi splittings are ≈300meV for both X1 and X2. **b** Simulated transmission of a similar cavity without excitonic absorption in the CBP:BSBCz layer (empty cavity). **c** Measured transmission spectra at different angles for the same cavity as discussed in **a.**

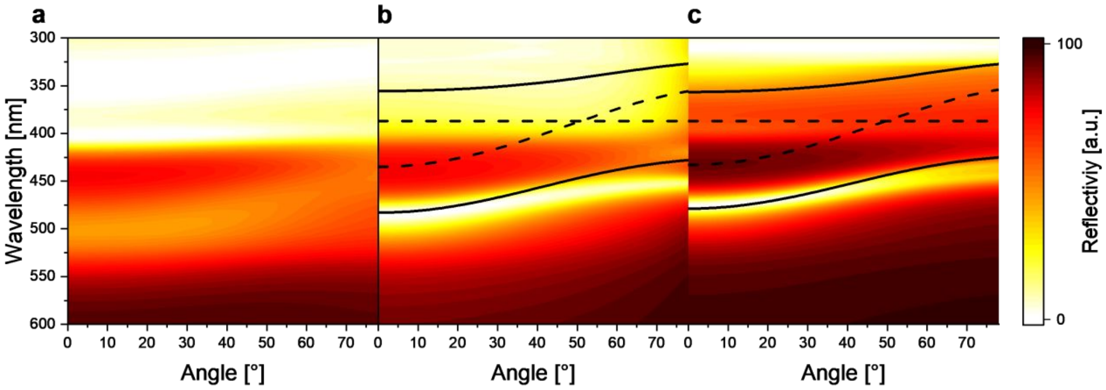

Figure S9: Simulations of the reflectivity spectra of **a** the reference OLED, **b** the BE-POLED, and **c** the TE-POLED. In b and c the exciton energy and the cavity mode are indicated as dashed lines and the upper and lower polariton branches as calculated by a coupled oscillator model are indicated as straight lines.
